# *Meloidogyne Haplanaria*: An Emerging Threat to Tomato Production in Florida

**DOI:** 10.2478/jofnem-2022-0032

**Published:** 2022-09-30

**Authors:** Lisbeth Espinoza-Lozano, S. Joseph, W. T. Crow, J. Noling, T. Mekete

**Affiliations:** 1 Escuela Superior Politécnica del Litoral, ESPOL, Facultad de Ciencias de la Vida Campus Gustavo Galindo Km., Guayaquil, Ecuador; 2 Entomology and Nematology Department, University of Florida, Gainesville, FL 32611 US; 3 Citrus Research and Educational Center, University of Florida, Lake Alfred, FL 33850 US

**Keywords:** interaction, *Meloidogyne haplanaria*, *Mi-*gene, resistance, tomato

## Abstract

The *Mi-*gene is widely used in different tomato cultivars to resist several Meloidogyne spp. (root-kot nematode; RKN), including *M. incognita, M. javanica*, and *M. arenaria*. Tomato cultivars with the *Mi*-gene are widely used in fields. However, factors such as temperatures, high initial population densities, and gene dosage can interfere with the expression of this gene. In addition, the presence of virulent species of RKN can limit the usefulness of the gene. One of the virulent species is *M. haplanaria*, which was identified infecting RKN-resistant tomato in Florida in 2015. The objectives of this study were to determine the initial damage threshold of *M. haplanaria* on tomato under greenhouse conditions and to analyze the impact of temperature and genetic background on virulence in tomato cultivars. The results showed a preliminary damage threshold of three eggs and J2/cm^3^ of soil. In addition, it was observed that *M. haplanaria* has a shorter life cycle than the virulent *M. enterolobii* and can infect, reproduce, and damage homozygous or heterozygous RKN-resistant tomato plants. This research demonstrated that *M. haplanaria* should be considered highly virulent on RKN-resistant tomato and is an important threat to agriculture in Florida.

Root-knot nematodes (RKN; *Meloidogyne* spp.) can cause severe damage to a wide variety of crops, including trees, vegetables, agronomic crops, turfgrasses, and ornamentals. The global annual crop lost to *Meloidogyne* spp. is estimated to be US$ 157 billion (Abad *et al*., 2008). *Meloidogyne incognita*, *M. javanica*, *M. arenaria*, and *M. hapla* are considered the most important RKN species worldwide, given their wide host range and their distribution around the globe (Moens *et al*., 2009). Some of these species are especially important to tomato plants, and they can cause up to 100% of yield losses (Seid *et al*., 2015).

*M. haplanaria*, or the Texas peanut RKN, was first described in Texas in 2003. This nematode was subsequently detected in Arkansas in 2016 (Khanal *et al*., 2016). The host range of this nematode includes tomato, pepper, legumes, radish, Indian hawthorn, ash, oak, cherry, maple, willow, rivercane, elm, bermudagrass, and birch. It was reported that the resistance conferred by the *Mi-*gene in tomato might not be effective against this nematode (Bendezu *et al*., 2004). The *Mi*-gene was initially found in *Lycopersicon peruvianum*, a wild type of tomato, and incorporated into commercial cultivars (Roberts, 1995). This source of resistance has been bred into commercial cultivars for several decades. However, it is increasingly common to observe virulent populations resulting from complex interactions among plants, nematodes, and environments (Davies and Elling, 2015). In Florida, the infection of *Mi*-gene-resistant plants by the RKN *M. enterolobii* already occurs (Brito *et al*., 2007). The detection of *M. haplanaria* on resistant tomato in Florida (Joseph *et al*., 2016) adds another barrier to adopting *Mi*-gene cultivars in this important tomato-producing state.

The objectives of this study were to (i) to determine the effect of different initial population densities of *M. haplanaria* on susceptible and resistant tomato cultivars “Rutgers” and “Sanibel”; (ii) to evaluate the impact of temperature on the stability of the *Mi*-gene and compare the different developmental processes of *M. haplanaria*, *M. incognita*, and *M. enterolobii*; and (iii) to compare the response of *Mi-*gene-resistant cultivars and rootstocks with the infestation of *M. haplanaria*, *M. incognita*, and *M. enterolobii*.

## Materials and Methods

### Plant material and inoculum preparation

The following procedures were used throughout all the experiments; only the tomato cultivars and nematode species differed among the different experiments. Tomato seeds were sown on Miracle-Gro Potting Mix (Scotts Miracle-Gro, Marysville, OH); maintained in a growth chamber for 4 wk at **~**26**°**C, 85% of humidity, and 12-hr light period; and watered daily. The population of *M. haplanaria* was initially identified using the protocol described by Joseph *et al*. (2016). Pure cultures were grown on susceptible “Rutgers” tomato (Burpee, Warminster, PA) and maintained in greenhouses on the University of Florida campus in Gainesville, FL. Nematode eggs were extracted from well-infested tomato roots using the method described by Hussey and Barker (1973).

### Tomato plant response to different initial population densities

Four-week-old tomato seedlings of resistant “Sanibel” (Reimer Seeds; Saint Leonard, MD) and susceptible “Rutgers” were transplanted onto 15.2-cm-top diameter clay pots filled with 1,000 cm^3^ of sandy loam soil previously autoclaved at 121°C for 30 min. *M. haplanaria* was inoculated onto plants at rates of 0, 0.25, 1, 2, 4, 8, 16, 32, and 64 eggs and second-stage juveniles (J2) per cm^3^ of soil by making four 3-cm-deep holes around the pot and discharging nematode egg solution through each hole. The pots were arranged on greenhouse tables using a randomized block design, with eight replicates per treatment. The tomato seedlings were planted into the clay pots 48 hr after the inoculation with the different nematode populations and maintained at the greenhouse at temperatures of ~28°C. The plants were watered daily and fertilized 20 days after transplanting with 2.5-cm^3^ Osmocote Smart-Release Plant food Plus (15-9-12, N-P-K) (Scotts Miracle-Gro, Marysville, OH) per pot. This experiment was repeated one time.

After 60 days, plants were uprooted, and shoots were cut at soil level, and height and fresh weight were recorded. Then, the roots were gently washed with running tap water and kept on a paper towel for 1 hr. The root length measurement was considered from the crown to the tip of the main root. The root gall index (GI) was assessed using the rating scale of 0 to 10, as described by Zeck (1971). The roots were weighed, and the total number of egg masses was counted after staining the whole root with 0.0015% phloxine B for 20 min at room temperature (Daykin and Hussey, 1985). Eggs were extracted using the method described by Hussey and Barker (1973).

The eggs were counted from 1-ml aliquot of egg suspension under an inverted microscope (Olympus CK30; Center Valley, PA) at a magnification of 40**×**. The reproduction factor (Rf) was obtained from the division of the final population by the initial population (Sasser *et al*., 1984). Data were analyzed according to the general lineal model (GLM), and if required, treatment means were separated according to Tukey’s HSD test (*P* **≤** 0.05) using SAS 9.1.3 software (SAS Institute, Cary, NC) and R studio (RStudio, Boston, MA) for running the Seinhorst model. The number of egg masses, eggs, eggs per gram of root, and root GI were regressed on the initial population for each tomato cultivar. Rf was regressed on the initial population. The Seinhorst model y **=** m **+** (1 – m) z P-T (Seinhorst, 1965) was fitted to the shoot weight, shoot height, root weight, and root length data in R. In this model, “y” is the relative yield of the evaluated parameters (the ratio between the yield at a given Pi and the average yield at Pi **£** T, with y **=** 1 at Pi **£** T), m is the minimum relative yield (the value of y at very large Pi), P (**=** Pi) is the initial nematode population density at the time of transplanting, and z is a constant **<** 1, with z -T **=** 1.05.

### Impact of temperature on the stability of *Mi*-gene in tomato plants

Tomato plants of Sanibel and Rutgers and nematode populations of *M. incognita*, *M. enterolobii*, and *M. haplanaria* were used in this experiment. Four-week-old tomato seedlings were transplanted into 3.1-cm-top diameter and 21.6-cm-deep Cone-Tainers (Stuewe and Sons, Tangent, OR) filled with 120 cm^3^ of autoclaved sandy loam soil. After 48 hr, each cone was inoculated with 360 eggs and J2, and the cones were placed on the racks in a completely randomized design. The cones of each treatment were placed in separate temperature-controlled growth chambers at 24°C, 28°C, and 32°C and maintained at 60% of relative humidity and 14-hr photoperiod. The plants were watered daily and fertilized 20 days post-inoculation with 20 cm^3^ of Miracle-Gro All Purpose Plant Food (24-8-16; N-P-K) (The Scotts Company, Marysville, OH). Forty days after inoculation, the plants were harvested; data on the root GI were assessed using the rating scale described by Zeck (1971). Plant roots were cleared, and egg masses were stained using the acid fuchsin method, as described by Byrd *et al*. (1983). Eggs were extracted using the method described by Hussey and Barker (1973). The number of eggs, J2/g, J3/g, and J4/g of root, was counted based on the differences in developmental stages described by Moens *et al*. (2009). This experiment was repeated one time.

Data were analyzed using SAS 9.1.3 software. In order to compare nematode development and infectivity assessment among species, data were log (*x* **+** 1)-transformed for analysis to fulfill the criteria for normality, and treatment means were separated according to Tukey’s HSD test (*P* **≤** 0.05). The response of *M. haplanaria* to temperature was evaluated by regressing total egg production, the number of egg masses, eggs per gram of root, and root GI on temperature for each tomato cultivar.

### Comparative response of *Mi*-resistant cultivars and rootstocks to *M. haplanaria*, *M. incognita*, and *M. enterolobii*

The relative responses of resistant tomato cultivars Sanibel and “Amelia” (Harris Seeds, Rochester, NY); the resistant rootstocks “Estamino” (Johnny’s Selected Seeds, Winslow, ME) and “Maxifort” *(MiMi)* (Johnny’s Selected Seeds, Winslow, ME); and the susceptible Rutgers and “Monica” (*mimi*) (Johnny’s Selected Seeds, Winslow, ME) to *M. haplanaria*, *M. incognita*, and *M. enterolobii* were evaluated. Nematode inoculation was performed on clay pots of 15.24 cm top diameter filled with 1,000 cm^3^ of autoclaved sandy loam soil. Each pot was inoculated, making four 3-cm-deep holes in the soil, and using a pipet, a solution containing 3,000 eggs and J2 was evenly discharged through the holes. After 48 hr, 4-wk-old seedlings from each tomato cultivar or rootstock were transplanted into the pots and maintained in a greenhouse with temperatures of 28°C ± 2, 12-hr photoperiod, and ~60% of relative humidity. The plants were fertilized 20 days after transplanting with 3 g of Osmocote Plus Smart-Release Plant Food per pot. Data were collected following the same methodology, as described for the previous experiment. This experiment was repeated one time. All count data were analyzed using the statistical software SAS 9.1.3. Proc GLM was used to determine the difference between the treatments. Nematode reproductive parameters and root infection were addressed by counting the different juvenile stages inside the roots, and the data were analyzed using a logarithmic transformation (log x + 1) of the counting data to fulfill the criteria for normality. Treatment means were separated according to Tukey’s HSD test (*P* < 0.05).

## Results

### Tomato plant response to different initial population densities

The data sets from the two experiments did not differ among them (*P* **>** 0.05) on all the evaluated parameters; therefore, the data were combined for analysis. *M. haplanaria* was able to reproduce at all initial population densities on both Sanibel and Rutgers tomatoes.

**Figure 1:**
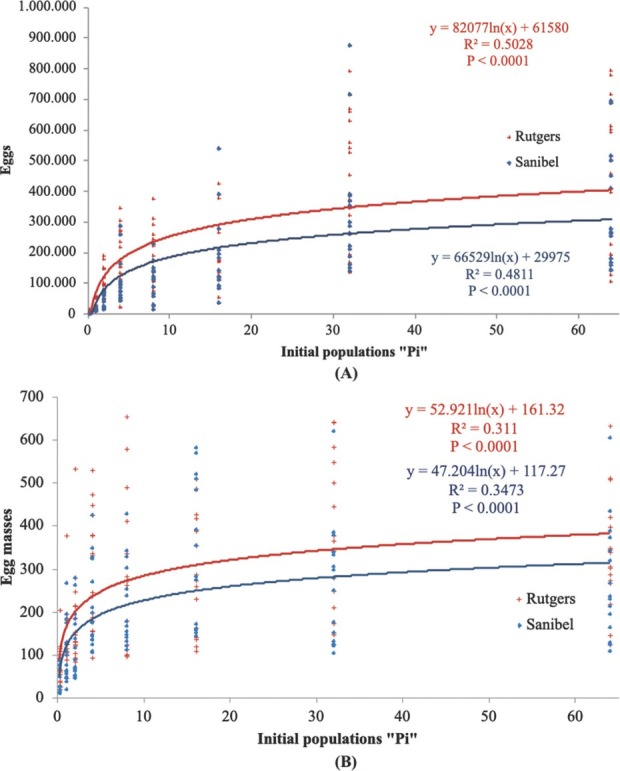
Regression of (A) total eggs and (B) egg masses on the initial population density of *Meloidogyne haplanaria* for tomato cultivars Rutgers (susceptible) and Sanibel (resistant), 60 days after inoculation with 0, 0.25, 1, 2, 4, 8, 16, 32, or 64 eggs and J2/g of soil under greenhouse conditions.

**Figure 2:**
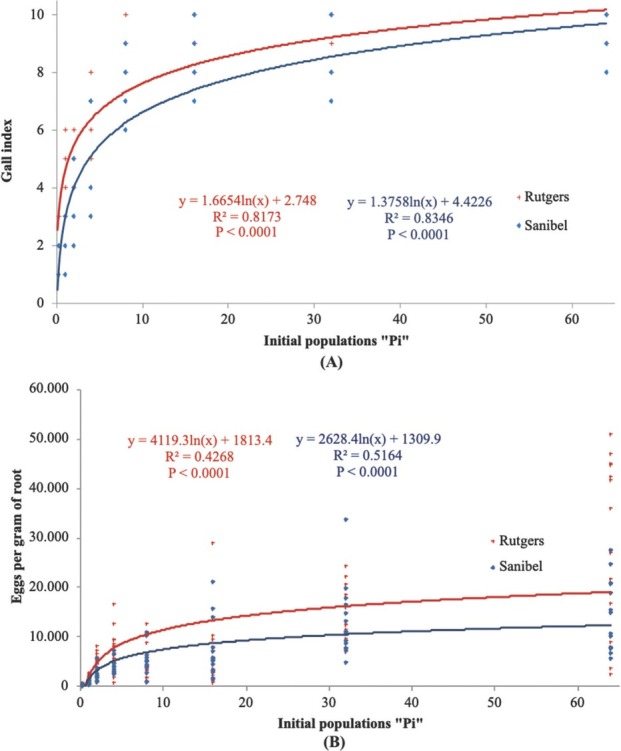
Regression of (A) GI and (B) eggs per gram of roots on the initial population density of *Meloidogyne haplanaria* for tomato cultivars Rutgers (susceptible) and Sanibel (resistant), 60 days after inoculation of 0, 0.25, 1, 2, 4, 8, 16, 32, or 64 eggs and J2/g of soil in greenhouse conditions. GI, gall index.

**Figure 3:**
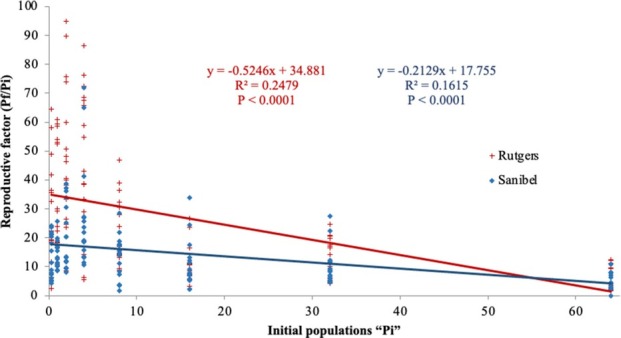
Regression of reproductive factor on the initial population density of *Meloidogyne haplanaria* for tomato cultivars Rutgers and Sanibel, 60 days after inoculation of 0, 0.25, 1, 2, 4, 8, 16, 32, or 64 eggs and J2/g of soil in greenhouse conditions.

Tomato cultivars and initial population densities (*Pi*) had a significant effect (*P ≤* 0.05) on reproductive parameters. Reproductive parameters, egg masses, total eggs, root GI, eggs per gram of root, and Rf were significant at *P* < 0.0001 for Rutgers and Sanibel. The regression analysis of the reproductive parameters (Figs. 1,2) was fitted on a logarithmic model, making it possible for observing a separation on the curves of Rutgers and Sanibel. The curves start flattening around initial populations of 32 eggs and J2/cm^3^ of soil. The regression analysis on the reproductive factor (Fig. 3) shows that reproduction in Rutgers was high compared with Sanibel at the lowest initial population density (0.25 eggs and J2/cm^3^) (*P* < 0.0001). The regression analysis presented a negative slope for both Rutgers and Sanibel (–0.5246; –0.2129).

**Figure 4:**
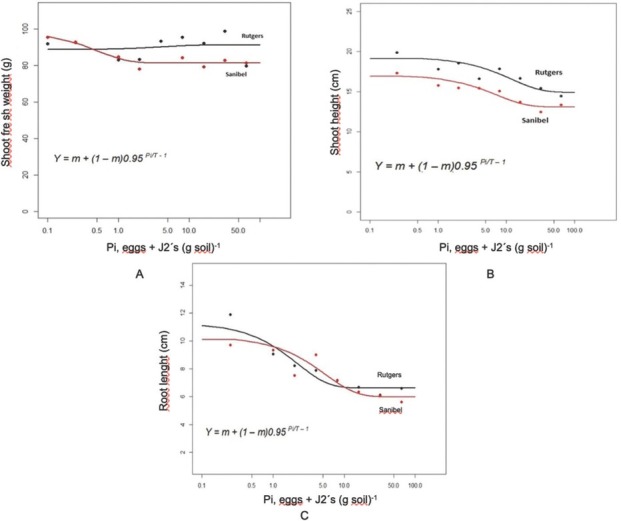
Relationship between the initial population density (Pi) of *Meloidogyne haplanaria* and (A) shoot fresh weight, (B) shoot height (cm), and (C) root length on tomato cultivars “Rutgers” and “Sanibel”. Plants were harvested after 60 days, and each point in the graph represents a mean of 16 replications, and the line is the predicted function obtained when the data were fitted to the Seinhorst model. The parameters obtained for were (A) for Rutgers: Y **=** 64.65; m **=** 0.15; T **=** 7.9; and for Sanibel Y **=** 66.27; m **=** 0.83; T **=** 0.64, (B) for Rutgers: Y **=** 64.64; m **=** 0.14; T **=** 3.25; and for Sanibel Y **=** 66.27; m **=** 0.83; T **=** 3.14, (C) for Rutgers: Y **=** 24.02; m **=** 14.55; T **=** 0.9; and for Sanibel Y **=** 24.86; m **=** 13.74; T **=** 1.2.

For shoot weight, the Seinhorst model was fitted only to Rutgers (Fig. 4A), but for shoot height (Fig. 4B) and root length (Fig. 4C) was fitted to both Rutgers and Sanibel. The tolerance limit (T) was determined at 1 and 3 eggs and J2/cm^3^ of soil for root length and plant height parameters, respectively, for both Rutgers and Sanibel. However, the model did not fit well for root weight of either cultivar (not shown).

### Impact of temperature on the stability of *Mi*-gene

The data sets from the repetitions did not differ statistically on the parameters considered for this experiment (*P* **>** 0.05); thus, the data from the repetitions were combined for analysis. Visual observations of Rutgers and Sanibel maintained at 24**°**C and 28**°**C showed normal growth, whereas plants that were kept at 32**°**C expressed symptoms of heat stress such as stunting, wilting, necrosis, and reduced leaf area.

**Figure 5:**
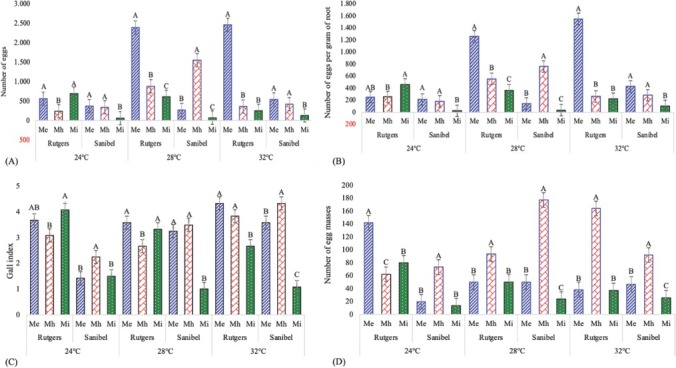
Effects of temperature on (A) total number of eggs, (B) eggs per gram of root, (C) GI, and (D) total egg masses on tomato varieties Rutgers and Sanibel inoculated with *Meloidogyne enterolobii* (Me), *M. haplanaria* (Mh), or *M. incognita* (Mi) 40 days after inoculation in growth chambers maintained at 24^°^C, 28^°^C, and 32**°**C. Columns within the same cultivar and at the same temperature with common letters are not different (*P* **≤** 0.05) according to Tukey’s test. GI, gall index.

The number of nematode eggs, egg masses, eggs per gram of root, and root GI differed among nematode species (*P* < 0.0001) and were affected by both host cultivar and temperature. On the susceptible cultivar Rutgers, total egg production and eggs per gram of root were highest for *M. incognita* at 24**°**C, while those were highest for *M. enterolobii* at 28**°**C and 32**°**C. However, on the resistant cultivar, for Sanibel, the total egg production and eggs per gram of root were lowest for *M. incognita* at all temperatures. At 24**°**C, *M haplanaria* and *M. enterolobii* were not different from each other. Egg production and eggs per gram of root for *M. haplanaria* were greatest at 28**°**C, while those of *M. enterolobii* were greatest at 32**°**C (Figs. 5A,B). On the susceptible Rutgers at 24**°**C, *M. enterolobii* had the greatest number of egg masses, and *M. haplanaria* had the least, while at 28**°**C and 32**°**C, *M. haplanaria* had the most egg masses, and *M. enterolobii* and *M. incognita* were not different from each other. On the resistant Sanibel, *M. haplanaria* produced the greatest, *M. enterolobii* intermediate, and *M. incognita* the lowest number of egg masses at all temperatures (Fig. 5C). On the susceptible Rutgers, the root GI was lowest for *M. haplanaria* at 24**°**C and 28**°**C but lowest for *M. incognita* at 32**°**C. On the resistant Sanibel, the root gall index was least for *M. incognita* at all temperatures and greatest for *M. haplanaria* at 24**°**C and 32**°**C (Fig. 5D).

**Figure 6:**
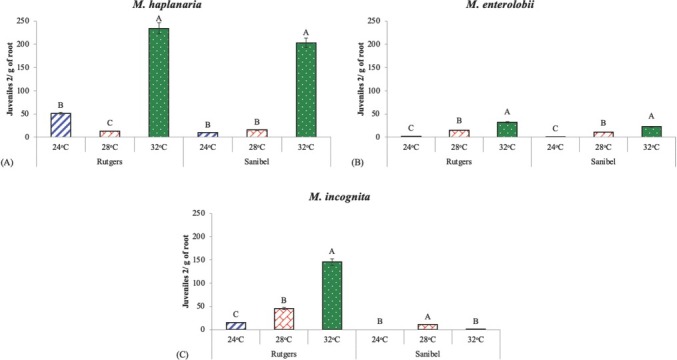
Effect of temperature on the total number of J2s/g of root observed within tomato roots of cultivars “Rutgers” and “Sanibel” 40 days after inoculation with (A) *Meloidogyne enterolobii*, (B) *M. haplanaria* or, and (C) *M. incognita* in a growth chamber maintained at 24**°**C, 28**°**C, and 32**°**C. Columns within the same cultivar with common letters are not different (*P* **≤** 0.05) according to Tukey’s test.

The J2/g of root differed with nematode species, host cultivar, and temperature. The number of *M. haplanaria* and *M. enterolobii* J2 within roots was greater at 32**°**C on both tomato cultivars, whereas *M. incognita* J2 numbers in roots were greatest on Rutgers at 32**°**C (Fig. 6). The number J3s and J4s/g of root were not significant (*P* **>** 0.05) for any of the treatments and cultivars (data not shown).

### Response of *Mi*-resistant cultivars and rootstocks to *M. haplanaria*, *M. incognita*, and *M. enterolobii*

The data from the two experiments did not show significant differences among them on all the evaluated parameters (*P* **>** 0.05); consequently, the data from the repetitions were combined for analysis. The RKN species *M. enterolobii, M. haplanaria*, and *M. incognita* reproduced on all cultivars and rootstocks. Differences among cultivars were observed in total egg masses, total eggs, root GI, and eggs per gram of root for each nematode species (*P* < 0.0001).

**Figure 7:**
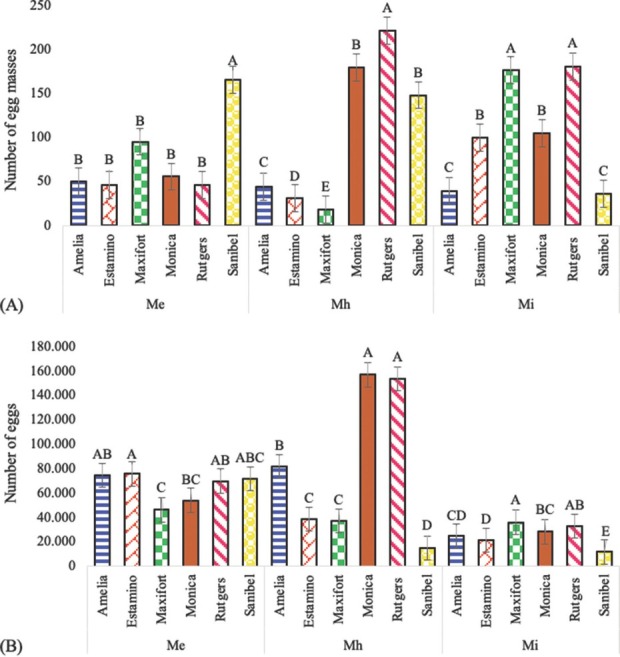
Effects of *Meloidogyne enterolobii* (Me), *M. haplanaria* (Mh), and *M. incognita* (Mi) on the number of (A) egg masses and (B) total eggs on the tomato cultivars “Amelia”, “Estamino”, “Maxifort”, “Monica”, “Rutgers”, and “Sanibel” 60 days after inoculation under greenhouse conditions. Columns within the same cultivar with common letters are not different (*P* **≤** 0.05) according to Tukey’s test.

Across the experiment, a range of 8 to 422 egg masses within the entire root system were observed. Among the cultivars inoculated with *M. enterolobii*, Sanibel presented the greatest number of egg masses. On the other hand, the greatest number of egg masses for *M. haplanaria* occurred in Rutgers and the least in Maxifort. *M. incognita* had the greatest number of egg masses on the susceptible control Rutgers and Maxifort and the least on the resistant Sanibel and Amelia (Fig. 7A).

The total number of eggs ranged from 7,200 to 208,800 and differed among the cultivars when inoculated with *M. enterolobii* (*P* = 0.0009), *M. haplanaria*, and *M. incognita* (*P* **<** 0.0001). Tomato cultivars inoculated with *M. enterolobii* presented minor differences among the cultivars; Maxifort presented the lowest number of eggs. Conversely, in cultivars infected with *M. haplanaria*, the largest number of eggs was observed in Monica and the susceptible cultivar Rutgers, whereas the lowest was reported in Sanibel. In response to inoculations with *M. incognita*, Maxifort and Sanibel had the highest and the lowest number of eggs, respectively (Fig. 7B).

**Figure 8:**
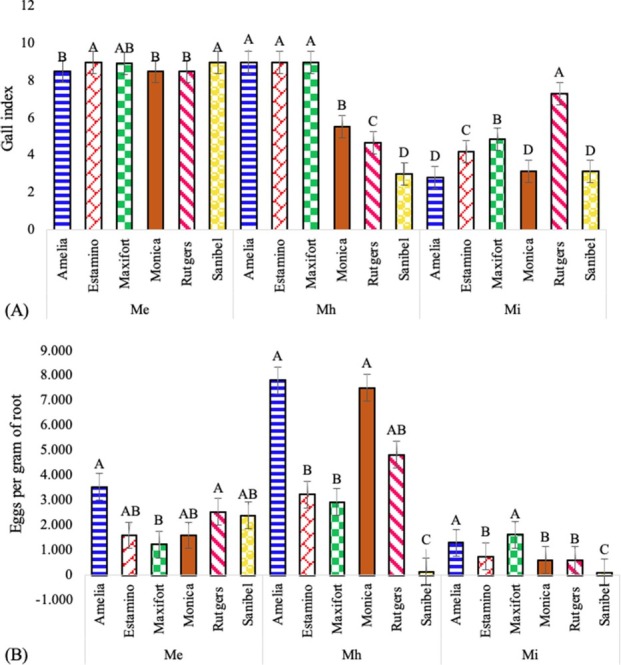
Effect of *Meloidogyne enterolobii (Me)*, *M. haplanaria (Mh)*, and *M. incognita (Mi)* on the number of (A) GI and (B) eggs per gram of root in the tomato cultivars “Amelia”, “Estamino”, “Maxifort”, “Monica”, “Rutgers”, and “Sanibel” 60 days after under greenhouse conditions. Columns within the same cultivar with common letters are not different (*P* **≤** 0.05) according to Tukey’s test. GI, gall index.

The GI for the infected plants was between 2 and 9 on a scale of 0 to 10. The GI was highly affected by nematode species, *M. enterolobii*, *M. haplanaria*, and *M. incognita* (*P* **<** 0.0001). All the cultivars inoculated with *M. enterolobii* presented a GI between 8 and 9. Inoculations of the cultivars with *M. haplanaria* produced the greatest GI on Amelia, Estamino, and Maxifort and the least on Sanibel (Fig. 8A). The cultivar Rutgers inoculated with *M. incognita* showed the largest GI, whereas the lower GI was reported in Amelia (Fig. 8A).

The number of eggs per gram of root was different among the cultivars, ranging from 1.81 to 4.52. Plants infected with *M. enterolobii* had variations in the number of eggs per gram root; the cultivar Amelia presented the largest number of eggs per gram of root, whereas Maxifort had the lowest number. When cultivars were inoculated with *M. haplanaria*, a larger infestation was reported in Amelia and Monica, whereas lower results were observed in Sanibel. Inoculations with *M. incognita* resulted in a low production of eggs per gram of root in cultivar Sanibel; meanwhile, Maxifort had a high production of eggs (Fig. 8B). In addition, J2 were counted at 32**°**C to observe differences in nematode life stages. Rutgers and Sanibel had a production of 32.2 J2/g and 22.9 J2/g of root, respectively, when they were inoculated with *M. enterolobii*, while *M. haplanaria* generated 234.46 J2/g of root in Rutgers and 203.23 J2/g of root in Sanibel. In addition, at 32**°**C, *M. incognita* produced 145.28 J2/g of root in the susceptible cultivar Rutgers and 0.68 J2/g of root in the resistant cultivar Sanibel.

## Discussion

Reproduction of *M. haplanaria* was observed to increase in both tomato cultivars Rutgers and Sanibel, indicating that both tomato cultivars are suitable hosts for *M. haplanaria* and suggesting that plant damage was correlated with a high initial population density and reproductive output. Root gall severity, the number of egg masses per root system, and the total number of eggs per root system were increased with the initial inoculum level of *M. haplanaria*, indicating their virulence on the tested tomato cultivars. This result agrees with several other studies (Eisenback *et al*., 2003; Bendezu *et al*., 2004; Joseph *et al*., 2016).

The Seinhorst model was fitted for plant height and root length against Pi for both Rutgers and Sanibel, but shoot weight was fitted only for Rutgers. The tolerance limit (T) was one nematode/cm^3^ and three nematodes/cm^3^ soil for root length and plant height parameters for both Rutgers and Sanibel, indicating that these cultivars are not suitable to be planted in *M. haplanaria*-infested areas. Nematode growth parameters were more informative and consistent across the initial population densities and cultivars. Rutgers allowed greater nematode reproduction than Sanibel, as expected. As the population increased, there was also an increase in the number of egg masses and total eggs per root system, GI, and eggs per gram of root. These results indicate the overcoming of the *Mi* resistance in Sanibel plants by *M. haplanaria*. It was also observed that the carrying capacity for the nematode infection was around 32 eggs J2/cm^3^ of soil. Inserra *et al*. (1983) demonstrated the different tolerance levels for susceptible and resistant cultivars of alfalfa to *M. hapla* and determined the tolerance limit in 1.6 eggs/cm^3^ and 7 eggs/cm^3^ of soil for the susceptible and resistant cultivars, respectively. Additional studies determined different tolerance levels depending on the nematode species and the type of experiment (pot, greenhouse, field, etc.) (Di Vito and Ekanayake, 1984; Di Vito *et al*., 1991). Therefore, the data collected in this study provided a preliminary tolerance value to be used for future microplots or field experiments. In addition, the Rf for both cultivars showed a negative slope across the different population densities, meaning that the Rf decreased as the initial population densities increased. Rutgers presented an Rf of 27.9, whereas Sanibel had 13.82 under greenhouse conditions. Host suitability of susceptible and resistant cultivars in net cage and microplot conditions demonstrated that Rf decreased as the initial populations increased. The same study also reported greater Rf in microplot experiments than in the net cage experiment (Fourie *et al*., 2010).

Rutgers and Sanibel were both negatively impacted by a constant temperature of 32**°**C. In addition, the temperature had a profound effect on nematode development and reproduction, although differences among nematode species and tomato cultivars were observed. *M. enterolobii* is considered a highly virulent species of RKN (Kiewnick *et al*., 2009), and it was able to reproduce on both Rutgers (susceptible) and the resistant Sanibel. Our experiment presented greater values of total eggs, GI, and eggs per gram of root of *M. enterolobii* in Rutgers than in Sanibel. Juanhua *et al*. (2013) reported an increase in reproduction parameters of *M. enterolobii* at temperatures of 28**°**C and 30**°**C. Similar results were observed on both resistant Sanibel and Rutgers at the respective temperatures. With a two-degree increase (32**°**C), we observed a reduction in the total egg mass per root system for Rutgers. This result could be due to the response of the nematode to higher temperatures or the plants enduring severe heat stress.

Information on the effects *M. haplanaria* has on the resistance *Mi*-gene in different tomato varieties and plant physiological response is lacking. Bendezu *et al*. (2004) reported that *M. haplanaria* was able to reproduce in Rutgers and “Motelle” successfully. Motelle carried the *Mi*-gene and was observed to be resistant to *M. arenaria* at air temperatures lower than 28**°**C, with a GI of 3.2 in Rutgers, and a GI of 3.5 was observed with *M. haplanaria*. Our results indicate similarities at a constant 24**°**C for Rutgers, whereas resistant Sanibel presented a slightly lower GI at the same temperature. However, as the temperature increased, the root GI also increased.

Observations on roots and counts of different nematode developmental stages at temperatures of 24°C, 28°C, and 32°C at 40 days after inoculation have shown significant differences in the response of cultivars Rutgers and Sanibel inoculated with *M. enterolobii*, *M. haplanaria*, and *M. incognita*. Similar results were observed on the life cycle of *M. hapla* on lettuce at different temperatures, where temperature regimes of 26.0°C to 32.2°C produced mature females 14 days after inoculation (Wong and Mai, 1973). Even though the total egg count was low for *M. haplanaria* in Sanibel at 32°C, plants exhibited extreme damage; this stress could be explained by the early hatching of eggs into J2 reinfesting the root system.

The response of Rutgers and Sanibel to infection by *M. incognita* agreed with previous findings, where higher reproduction was observed in Rutgers and lower reproduction in Sanibel (Brito *et al*., 2007). In addition, we found that the development of *M. incognita* was slower than that of *M. enterolobii* and *M. haplanaria*; in this study, the presence of *M. incognita* J3s and J4s were not observed at 32**°**C in Rutgers or Sanibel.

All the cultivars tested were highly susceptible to *M. enterolobii*. This affirmation was noticed particularly on the parameters GI and total eggs. Sanibel is a RKN-resistant and heat-tolerant cultivar and presented fewer eggs, GI, and eggs per gram of root when it was inoculated with *M. haplanaria* or *M. incognita*. Estamino and Maxifort are used as nematode-resistant rootstock, but our results showed that both were susceptible to the nematodes tested.

Maxifort is a cross from *Solanum lycopersicum* x *S. habrochaites* known to be a homozygous resistant cultivar (*MiMi*), but our experiment showed infection by *M. incognita*, *M. enterolobii*, and *M. haplanaria*. Cortada *et al*. (2009) evaluated the susceptibility of Maxifort against several RKN species, such as *M. javanica*, *M. arenaria*, and *M. incognita*, and reported that other homozygous crosses (*MiMi*) presented a reduced infection of the RKN. Other authors suggest that the homozygosis and heterozygosis of a cultivar have a direct effect on the gene expression and can interfere with the normal development of the nematode (Trudgill and Phillips, 1997; Tzortzakakis *et al*., 1998; Jacquet *et al*., 2005). From our study, the data were not conclusive in determining the resistance or tolerance of any of the evaluated cultivars to *M. haplanaria*. *Meloidogyne enterolobii* is known as a virulent root-knot species and can overcome the Mi resistance; our results indicate that *M. haplanaria* is also highly virulent and possibly more virulent than *M. enterolobii*. We found that none of the evaluated cultivars had resistance against any of the nematode populations tested. We hypothesize that the heat tolerance trait present in Sanibel may have a partial effect on the resistance of this cultivar to RKN. However, it is important to understand in detail how resistance processes and gene dosage directly interfere with the infection of new species of virulent nematodes such as *M. haplanaria* and how this information could help in the development of new cultivars with better and broader resistance to these virulent nematodes.

This study provided an initial insight into the effects of *M. haplanaria* and its potential development on tomato cultivars in Florida; it established an initial threshold under greenhouse conditions and determined that *M. haplanaria* produced a larger number of J2 during the same period in comparison to *M. incognita* and *M. enterolobii* at the same conditions. Finally, we found that *M. haplanaria* can infect and reproduce on all plant materials tested, regardless of their genetic background or resistant gene dosage. This research is expected to contribute to a better understanding of the effect of *M. haplanaria* on tomato crops and to serve as a baseline for additional studies under field conditions and thus avoid potential economic losses in this crop and other hosts.
